# Lake sturgeon (*Acipenser fulvescens*) growth and longevity estimated from adult capture–mark–recapture data

**DOI:** 10.1111/jfb.70478

**Published:** 2026-04-28

**Authors:** Edward A. Baker, Scott F. Colborne, Nancy A. Auer, Kim T. Scribner, Douglas L. Larson, Andrew S. Briggs, Ronald M. Bruch, Margaret H. Stadig, Michael C. Donofrio

**Affiliations:** ^1^ Michigan Department of Natural Resources Marquette Fisheries Research Station Marquette Michigan USA; ^2^ Quantitative Fisheries Center, Department of Fisheries and Wildlife Michigan State University East Lansing Michigan USA; ^3^ Department of Biology Michigan Technological University Houghton Michigan USA; ^4^ Michigan Department of Natural Resources Lake St. Clair Fisheries Research Station Harrison Township Michigan USA; ^5^ Wisconsin Department of Natural Resources Oshkosh Service Center Oshkosh Wisconsin USA; ^6^ Wisconsin Department of Natural Resources Peshtigo Wisconsin USA

**Keywords:** capture–mark–recapture, growth, lake sturgeon, longevity, survival

## Abstract

Effective fishery management policy depends on accurate life‐history data, particularly for harvested species. Longevity is a core life‐history trait that is directly related to annual survival and lifetime reproductive potential, but fish longevity is generally not well documented, particularly for long‐lived species. We used capture–mark–recapture data spanning 44 years as an alternative method to estimate sex‐specific adult lake sturgeon (*Acipenser fulvescens*) average annual growth and longevity for five populations in the Laurentian Great Lakes. We determined individual fish average annual growth and fit population and sex‐specific exponential lines for average annual growth as a function of fish length at initial capture. We used the exponential equations to estimate age for 180 cm fork length (FL) female and 160 cm FL male lake sturgeon from each population by sequential subtraction of annual growth increments and assuming age at onset of sexual maturity of 24 and 15 years for females and males, respectively. Average annual growth estimates varied between sexes and among populations and ranged from 0.26 to 1.15 cm year^−1^. Estimated ages also varied among populations. Age estimated for a 160 cm FL male lake sturgeon across the five populations ranged from 90 to 279 years. Age for a 180 cm FL female across populations ranged from 99 to 427 years. Our data suggest lake sturgeons live much longer than currently believed. High longevity and inferentially higher adult survival have important implications for sturgeons in general and support conservative harvest regulations for lake sturgeon fisheries.

## 1 INTRODUCTION

Accurate life‐history data are vital for setting effective fishery management policy and ensuring long‐term population persistence, particularly for harvested species (Young et al., 2006). Longevity is a core life‐history trait that is directly related to annual survival and lifetime reproductive potential. However, longevity is one aspect of fish life history that is generally not well understood (Das, 1994), particularly for long‐lived species. Key and covarying life‐history traits including number and size of offspring, growth rate and age‐specific survival rates are shaped by natural selection to maximize fitness in a given environment (Healy et al., 2019; Kolora et al., 2021). Longevity is known to vary widely among fish taxa, ranging from 59 days for adorned dwarfgoby (*Eviota sigillata*; Randall & Delbeek, 2009) to more than 400 years for Greenland shark (*Somniosus microcephalus*; Nielsen et al., 2016). Both longevity and survival rates (along with recruitment rates) influence population persistence, particularly when populations are subjected to harvest pressure (Boreman, 1997).

Populations of long‐lived organisms, including sturgeons, are particularly susceptible to increased mortality at any life stage, and increased adult mortality is particularly detrimental to population recovery and persistence (Boreman, 1997). The importance of accurate age and longevity estimates for long‐lived species was highlighted in analyses of Blandings turtle (*Emydoidea blandingii*) demographics. Congdon et al. (1993) showed that population recovery in Blandings turtles was significantly constrained by life‐history traits that make them slow to respond to increased mortality and subsequent population declines. The historical decline of sturgeon across the northern hemisphere and the lack of recovery for most populations are due to anthropogenic factors, particularly harvest, that resulted in increased adult mortality, and life‐history traits, including longevity, late maturity, intermittent spawning and low natural recruitment rates, that make population recovery a slow process (Peterson et al., 2007).

Longevity in fishes has primarily been estimated from survival time in captivity (Randall & Delbeek, 2009) or from age estimated using annuli counts from bony or related structures (e.g., scales, otoliths, fin‐rays or spines) from wild fish (Das, 1994). Determining longevity for wild fish is challenging due to difficulties related to accurate age determination, particularly for relatively long‐lived fishes like sturgeons (Bruch et al., 2009; Withers et al., 2020). The accuracy by which fish longevity can be estimated depends on the reliability with which the age of individuals can be determined (Beverton, 1987). Accurate age and longevity data are important because longevity is related to annual survival rate. Underestimating a species longevity can lead to underestimation of annual survival (Kerns & Lombardi‐Carlson, 2017) and consequent mismanagement of species subjected to harvest fisheries. The unregulated commercial harvest of lake sturgeon in the Great Lakes from the 1880s until 1920s and their continued low abundance since that time reflects this lack of knowledge about fundamental aspects of lake sturgeon life history.

Longevity in fishes is thought to be related to maximum adult size, with larger species reaching greater longevity (Randall & Delbeek, 2009). In addition to size, Beverton (1987) suggested longevity (and large size) in fishes has a phylogenetic basis (Healy et al., 2019). Specifically, phylogenetically basal (ancestral) species generally reach larger size and greater longevity. Sturgeons are an ancient group of fish that first appear in the fossil record in the Lower Jurassic, approximately 200 million years ago (Bemis et al., 1997). Sturgeons are also some of the largest extant freshwater and anadromous fish and are known for their longevity and size (Tsepkin & Sokolov, 1971).

Lake sturgeon is one of several exclusively freshwater members of Acipenseridae for which longevity is not well understood. Lake sturgeon longevity has been reported to be as high as 150 years (MacKay, 1963), but others have questioned whether this is typical for lake sturgeon or whether these ages represent the extreme (Pollock et al., 2015; Sulak & Randall, 2002). Lake sturgeon longevity is generally assumed to be less than the 150‐year estimate (Peterson et al., 2007). However, the 150‐year‐old lake sturgeon cited as the oldest on record was aged from a fin‐spine cross section (MacKay, 1963) and is likely an underestimate of the fish's true age (Bruch et al., 2009; Hessenauer et al., 2018).

Alternatively, fish longevity can be estimated based on growth rate increments determined from capture–mark–recapture data and comparing growth rate with maximum known size. Thorson and Lacy Jr (1982) used growth rate estimated from capture–mark–recapture data and age determined from vertebral annuli to estimate longevity of bull shark (*Carcharhinus leucas*). MacNeil et al. (2012) used capture–mark–recapture growth rate and maximum recorded size to conclude Greenland Sharks (*S. microcephalus*) may be among the oldest fish species. This conclusion was supported by subsequent work that used radiocarbon dating to estimate Greenland Shark longevity to be at least 272 years and potentially over 400 years (Nielsen et al., 2016).

Increased attention focused on lake sturgeon populations in the Laurentian Great Lakes region provided an opportunity to use growth rate estimates based on capture–mark–recapture data to improve estimates of longevity and understanding of lake sturgeon life history. Our objectives were to (1) use fish length measured at initial capture and marking, subsequent recapture, and years between captures to estimate mean annual growth rate for sexually mature male and female lake sturgeon; (2) apply mathematical models to describe sexually mature male and female lake sturgeon mean annual growth as a function of size at initial capture over the observed size range (including confidence intervals); and (3) estimate and compare sex‐specific lake sturgeon age and longevity based on growth rate models in geographically and environmentally distinct populations across the upper Laurentian Great Lakes.

## 2 METHODS

### 2.1 Ethics statement

Except for recent data collected from the Black Lake population, the data presented in this study were collected prior to widespread establishment of animal ethics committees. Furthermore, most of the data presented herein were collected by state government agencies that are not subject to animal ethics committee approvals. All fish capture and handling protocols did follow protocols outlined in Guidelines for the Use of Fishes in Research (Use of Fishes in Research Committee, American Fisheries Society, 2014). Since 2007, fish handling in the Black Lake system has been conducted under approved Michigan State University IACUC protocols 05/07‐056‐00, 03‐11‐048‐00, 03‐11‐049‐00, 03/14‐042‐00, 04/17‐060‐00, PROTO202000023 and PROTO202300067.

### 2.2 Study populations

**FIGURE 1:**
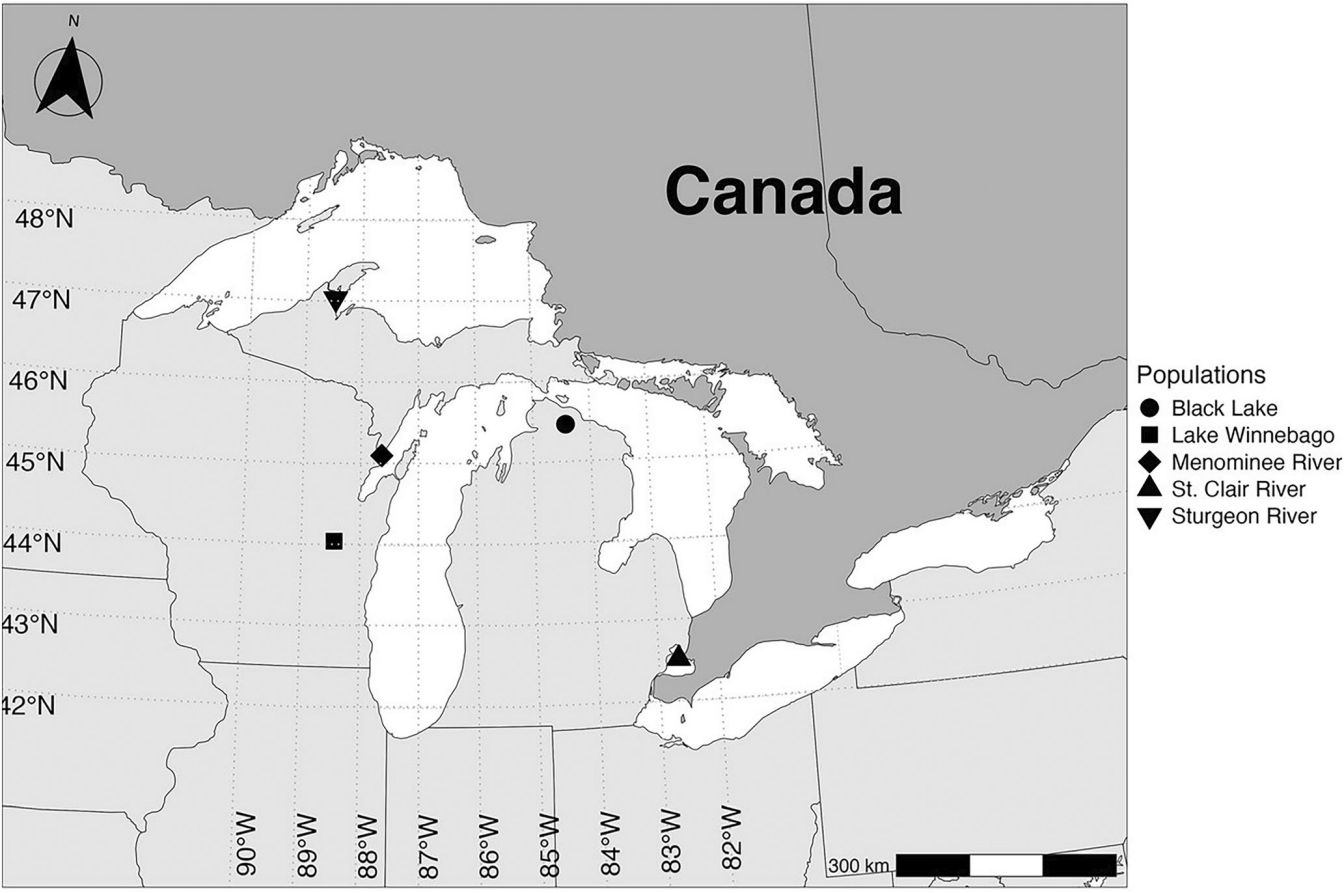
Map of Great Lakes showing approximate locations of sampled populations.

Five genetically distinct (DeHaan et al., 2006) and geographically separated lake sturgeon populations in the upper Laurentian Great Lakes including Black Lake, MI; Sturgeon River, MI; Menominee River, MI/WI; St. Clair River, MI/ON; and Lake Winnebago, WI (Figure 1) have been the subject of ongoing research or monitoring since at least 2000 (25+ years; details below). These populations are all native, self‐sustaining, and, except for Lake Winnebago, are at reduced abundance from historical levels (Holey et al., 2000).

Some recent stocking has occurred in the Black Lake, Menominee River, Lake Winnebago, and Sturgeon River populations (see below for details). However, the adult fish sampled for this study were likely naturally produced and initially captured prior to or shortly following the onset of stocking and before many stocked fish would have reached sexual maturity and been vulnerable to capture.

Black Lake is in the Cheboygan River drainage of the Lake Huron basin in Cheboygan County, Michigan, and supports an adult population estimated at 1183 as of 2022 (Larson et al., 2025). The Black Lake system is isolated from the Great Lakes by dams downstream of the lake. The system has been described previously by Baker and Borgeson (1999) and has been the subject of annual research and monitoring since 2001. Spawning fish have been sampled annually, and all captured fish have been tagged with passive integrated transponder (PIT) tags. There have been 608 unique individual females and 926 unique individual males captured, and 64% have been recaptured at least once since initial tagging. The minimum and maximum size of lake sturgeon included in analysis was 109–159 cm fork length (FL) for males and 127–172 cm FL for females. Four year classes of lake sturgeon were stocked in Black Lake in the mid‐1980s but the stocked fish were still immature and unlikely to be sampled when data collection began for the present study (Baker & Borgeson, 1999). All stocked fish were young‐of‐the‐year (YOY) and were not marked when stocked.

The Sturgeon River is a Lake Superior tributary in Baraga County, Michigan, and the study site has been previously described by Auer (1996). Spawning fish in Sturgeon River have free access to Lake Superior and have been sampled most years since 1987. From 1987 to 1999 several different external tags were used to mark fish including Floy, Monel, cattle ear and dart. Since 1999 fish have been PIT tagged and, in some years, also Floy tagged. Since 1999, 228 unique females and 689 unique males have been captured and tagged and 18% have been recaptured at least once. The minimum and maximum size of lake sturgeon included in analysis was 104–145 cm FL for males and 122–163 cm FL for females. YOY lake sturgeon were stocked in Sturgeon River in 1998, after data collection for the present study began. Stocked fish were not marked prior to stocking.

The Menominee River is a Lake Michigan tributary that forms the border between northeast Wisconsin and Michigan's Upper Peninsula and has been described previously by Thuemler (1985, 1997). The Menominee River is fragmented by multiple hydropower dams with limited upstream fish passage since 2015 (Isermann et al., 2022). Lake sturgeon monitoring has been ongoing since the late 1970s. Lake sturgeon sampling in the Menominee River has included a mixture of spawning assessments and summer and fall electrofishing surveys. Menominee River lake sturgeon have been tagged with PIT tags since 2000; prior to 2000 fish were tagged with external tags including Floy tags and Monel tags. Since 2000, a total of 7782 lake sturgeon have been captured and tagged, and sex has been assigned to 648 unique individual female and 1424 individual male lake sturgeon. Of these, 20.1% have been recaptured at least once. The minimum and maximum size of lake sturgeon included in analysis was 94–129 cm FL for males and 121–146 cm FL for females. The Menominee River was stocked with unmarked YOY and yearling lake sturgeon from 1982 to 1986, after sampling for the present study began. Furthermore, the fish were stocked in a reach of the river upstream of two hydroelectric dams from the reaches sampled for this study.

The St. Clair River is part of the St. Clair–Detroit River system (SCDRS), which also includes Lake St. Clair and the Detroit River. The SCDRS connects Lake Huron to Lake Erie and has been previously described by Chiotti et al. (2023). Lake sturgeon in the SCDRS system can range freely throughout the Great Lakes. Lake sturgeon monitoring in the St. Clair River has been ongoing since the late 1990s. Lake sturgeon sampling in the SCDRS has included a mixture of spawning assessments and summer surveys, but sampling in the St. Clair River has primarily been conducted using setline surveys around the spawning period. St. Clair River lake sturgeon have been tagged with PIT tags since 2001. Prior to 2001, fish were only tagged with external Monel tags as described by Briggs et al. (2019). Since 1996, a total of 2396 individual lake sturgeon have been captured and tagged in the St. Clair River, and 15.2% have been recaptured at least once. Of these fish, 1174 have been sexed using molecular sexing methods (Kanefsky et al., 2022; Scribner & Kanefsky, 2021), with 654 identified as female and 520 as male. The minimum and maximum size of lake sturgeon included in analysis was 113–147 cm FL for males and 126–165 cm FL for females.

The Lake Winnebago system is in the Lake Michigan drainage and is the largest inland lake system within Wisconsin. The system supports an adult lake sturgeon population estimated to be 41,353 fish as of 2004 (28,006 adult males and 13,347 adult females; Bruch, 2008). There are several locks and dams on the Fox River between Lake Winnebago and Lake Michigan, and lake sturgeon have been documented emigrating from Lake Winnebago to the Great Lakes. However, there are no dedicated fish passage facilities for upstream movement on the Fox River. Fish have been sampled annually during the open water season, including the spawning season, as well as during the tightly managed spearing season since the 1970s. All fish were checked for tags and any fish handled during the spawning season that did not have a tag was tagged with external tags prior to 1999 and with a PIT tag since 1999. Since the 1970s, 5807 unique females and 23,774 unique male lake sturgeon have been captured and tagged, and 49.4% have been recaptured at least once. The minimum and maximum size of lake sturgeon included in the analysis was 96–178 cm FL for males and 123–201 cm FL for females. Lake sturgeon were periodically stocked in Lake Winnebago beginning in 2002, after sampling for the present study began. Stocked fish were extended growth fingerlings or yearlings (>200 mm total length, TL), and were PIT tagged prior to stocking.

### 2.3 Data collection and analysis

#### 2.3.1 Sampling methods

Fish capture methods varied among rivers and seasons. In the Black River, the spawning habitat is a mix of relatively shallow wadable water and deeper sections that preclude wading. Personnel in Black River used wetsuits, masks and snorkels to dive and capture spawning fish using large dip nets, and fish were brought to shore for processing.

In Sturgeon River spawning fish were captured using large, long‐handled dip nets. In the Sturgeon River, the spawning habitat is shallow, and personnel were able to wade the spawning reach searching for fish. When observed, fish were netted and brought to shore for processing.

In the Menominee River, fish were captured using boat electrofishing and large dip nets. During spring spawning, electrofishing was focused on short river reaches immediately below dams where fish were known to congregate. During summer and fall surveys, electrofishing effort was spread out along the entire reaches of river between dams.

Lake sturgeon in the St. Clair River have been captured using a variety of gears, but fish included in this study were caught primarily with setlines deployed from a boat (Thomas & Haas, 1999). Lake sturgeon in the St. Clair River spawn in relatively deep water, making the sampling techniques used in the other study waterbodies unfeasible.

Lake sturgeon in the Winnebago system were captured using a variety of sampling gears including long‐handled dip nets during the spawning season, electrofishing during capture and transfer events in the spring and fall, and during the sturgeon spearing season when harvested fish were registered with WDNR staff.

#### 2.3.2 Fish measurement methods

Except for Lake Winnebago, captured fish were measured to the nearest 0.5 cm or 0.1 inch for TL, FL and girth, and some fish were weighed (lbs or kgs). Lake Winnebago fish were measured to the nearest 0.5 in from the 1970s to 2007 and have been measured to the nearest 0.1 in since 2008. Fish lengths recorded in inches were converted to cm for analysis. In the early years of monitoring fish in Menominee River and Sturgeon River fish were only measured for TL and Lake Winnebago system fish have only been measured for TL. TL measures were converted to FL using a linear regression equation developed from Black River data (*N* = 5223, *R* ^2^ = 0.98). Fish lengths were measured using either a rigid measuring board or a flexible measuring tape. When a flexible tape was used to measure fish the zero end of the tape was held in place at the tip of the snout and TL and FL were measured while the fish was on flat ground and the trunk of the fish was held in a straight line from snout to caudal fin. We used both the flexible tape and rigid measuring board on a subset (*N* = 172) of fish from Black River and compared methods using linear regression. We found no difference between tape and board length measurements (i.e., slope not significantly different from 1.0, *R* ^2^ = 0.994, *p* < 0.001). Field data were proofed for QA/QC and entered in either a database or spreadsheet for further analysis. Additional QA/QC included proofing entered data against field data sheets to insure accurate data entry.

#### 2.3.3 Sex and maturity determination

Lake sturgeon do not exhibit sexual dimorphism and can only be reliably sexed during spawning when fish are ripe and gametes are extruded (Vecsei et al., 2003) or via genetic sex determination (Scribner & Kanefsky, 2021). Black River and Sturgeon River fish were captured during spawning and were known to be sexually mature and reliably assigned sex based on expression of gametes. Menominee River, St. Clair River and Lake Winnebago fish were captured throughout the open water season, including during spawning, across years and many captured fish were either immature at initial capture or sexually mature but classified as unknown. Fish initially classified as unknown or immature were assigned sex when later recaptured during spawning, when registered during the winter spearing season on Lake Winnebago, or sex was determined by genetic analysis (Kanefsky et al., 2022; Scribner & Kanefsky, 2021). Because we focused on growth rate for sexually mature lake sturgeon, we only used data from fish classified as males that were 91 cm FL or larger at initial capture and females that were 120 cm FL or larger at initial capture for Black River, Sturgeon River and Menominee River fish, regardless of whether sex was determined at initial capture. These lengths approximate the smallest sexually mature fish captured during spawning in the Black, Menominee and Sturgeon rivers and so are approximate lengths at onset of sexual maturity for those populations. Because growth rate was greater for St. Clair River fish (Hessenauer et al., 2018), we used 104 cm FL for males and 122 cm FL for females as the length at onset of maturity. These lengths represent St. Clair River males aged 15 and females aged 24, when lake sturgeon reach maturity (Taylor et al., 2025). For Lake Winnebago males we used 95 cm FL and for females 122 cm FL for onset of maturity as these are approximate FL for 15‐year‐old and 24‐year‐old male and female lake sturgeon from Lake Winnebago (Bruch et al., 2009).

#### 2.3.4 Mean annual growth estimation

We calculated mean annual growth for individual lake sturgeon that were captured and subsequently recaptured at least 3 years after initial capture and for some fish up to 42 years between captures. Mean annual growth was calculated by subtracting the FL recorded at the initial capture event from FL recorded at the most recent recapture and then dividing by the number of years between capture events. Except for Lake Winnebago data, we used 3 years as the minimum time between captures because, based on a preliminary examination of growth data, this was the minimum span of time needed for lake sturgeon growth to be measurable when length was measured to the nearest 0.5 cm or 0.1 inch. For Lake Winnebago data we used 6 years as the minimum time between captures because Lake Winnebago fish were measured to the nearest 0.5 in prior to 2007.

We plotted population and sex‐specific individual fish mean annual growth rate as a function of FL at initial capture (Thorson & Lacy Jr, 1982) and fit an exponential line to the data. This approach is similar to the von Bertalanffy growth function (Fabens, 1965). The von Bertalanffy growth curve models a fish's size as a function of age and reaches an asymptotic maximum length. Our approach models an individual fish's mean annual growth rate as a function of size at initial capture, a surrogate of age and produces a curve that appears the inverse of a von Bertalanffy curve with an asymptotic growth rate. This assumes that relatively small, young fish grow at a faster rate than larger, older fish, an assumption also inherent in the von Bertalanffy growth model. Each observation in our data set represented a unique individual without repeated measures across multiple capture events. Therefore, we used a linear model framework, analysis of covariance (ANCOVA), rather than a generalized linear‐mixed effects model (GLMM). We fit an ANCOVA model using initial length as the covariate, sex and population as fixed factors, and the interaction between sex and population to test for growth differences among populations and between sexes.

To evaluate the relationship between fish length and annual growth, we modelled individual mean annual growth rate (G, in cm year^−1^) as an exponential function of initial FL, using the form:

(1) $$G=X\cdot e^{Z\cdot \mathrm{FL}},$$

where X is a scaling parameter (i.e., theoretical maximum growth rate)and *Z* controls the exponential rate of growth decline as initial fish size increases. As such, these two parameters are jointly part of estimating annual growth rates and are not fully independent of each other when estimating overall growth rates.

The growth relationship was evaluated using nonlinear models that were fit separately for each population and sex using the nls() function in R (version 4.2.2), applying nonlinear least squares (NLS) estimation. Initial values for *X* and *Z* were informed by population‐level fits derived during preliminary fitting of an exponential equation (see previous paragraph), but the final parameter estimates reflect the best statistical fit to the observed data. Standard errors for *X* and *Z* were obtained from the model summary output and used to quantify uncertainty and inform simulations. Model convergence was confirmed for all groups using default tolerance criteria, and residual standard errors were used to assess model fit. Statistical significance of the *X* and *Z* model parameters was evaluated using *t*‐tests associated with NLS estimation, which test the null hypothesis that parameter estimates do not differ from zero. These exponential growth models were then used to back‐calculate individual age estimates and to generate population‐ and sex‐specific age‐length prediction intervals.

#### 2.3.5 Age estimation

We used the exponential equation describing annual growth increment as a function of fish size to determine age (longevity) of a 160 cm FL male and 180 cm FL female from each population to permit comparison among populations. Age was estimated by sequentially subtracting the estimated annual growth increment in year *t* from the fish FL in year *t* to determine FL_*t*−1_ with the starting year (*t*) set at 2024. The form of the equation was

(2) $${\mathrm{FL}}_{t-1}={\mathrm{FL}}_{t}-\left(X^{*}\;e^{\left(Z^{*}\mathrm{FL}t\right)}\right),$$

where FL_*t*−1_ is fork length in year *t* − 1; FL_*t*_ is fork length in year *t*, and *X* and *Z* are derived coefficients (all lengths in cm). For example, a 180 cm FL female lake sturgeon in 2024 would be 179.5 cm FL in 2023 if the annual growth increment estimated from Equation (2) was 0.5 cm.

Sequential subtraction of annual growth increments was carried out until FL in year *t* was equal to FL at onset of sexual maturity for the sex and population. Onset of sexual maturity was set at 91 cm FL for males and 120 cm FL for females from the Black, Sturgeon and Menominee populations, 104 and 122 cm FL for St. Clair River males and females respectively, and 95 and 122 cm FL for Lake Winnebago males and females respectively. As noted above, these values are the approximate size at onset of sexual maturity for the populations examined. To determine year of hatch (*t*_*h*_), we then subtracted another 15 years from year *t* for males and 24 years from year *t* for females. Ages 15 and 24 are mean ages of first maturity for the sexes (Taylor et al., 2025). We then calculated age for a 160 cm FL male and a 180 cm FL female from each population by subtracting year of hatch from 2024. We used these lengths because they are approximate maximum lengths observed across populations and to allow direct comparison of estimated longevity among populations. We also estimated age of the largest observed individuals from each population as an estimate of maximum observed longevity for each population.

Uncertainty in growth parameters was incorporated into the age estimates by applying a bootstrap resampling procedure. Specifically, for each population and sex, we generated 1000 bootstrap datasets by resampling fish (with replacement) from the original data. For each bootstrap sample, we refit the NLS model to obtain new *X* and *Z* parameters. Using these parameters, we applied the recursive age estimation procedure to a standardized FL grid (90–205 cm, representing the observed breadth of fish lengths across populations and sexes). The resulting age estimates were summarized to provide the median age estimates at a given size and the 95% confidence intervals. These values were plotted as age‐length curves with uncertainty bands. Individual observed fish were also assigned estimated ages using the best‐fit model and plotted for comparison.

All calculations and analysis were completed using MS Excel and R version 4.2.2 (www.r-project.org).

## 3 RESULTS

### 3.1 Estimated mean annual growth rates

Population and sex‐specific sample sizes, FL ranges, and mean annual growth rates are summarized in Table 1. Initial FL of fish included in growth analysis ranged from 95 to 178 cm for males and 120 to 177 cm for females. Years elapsed between initial capture and most recent recapture ranged from 3 to 42 years.

**TABLE 1:** Sex‐specific sample sizes, size ranges, and mean annual growth estimates for adult lake sturgeon from five study populations and across all study years.

Population	Sex	N	Range of initial FL (cm) of fish included in analysis	Mean annual growth (cm ± 1 SE)
Black River	Male	58	109–159	0.37 ± 0.03
	Female	58	127–172	0.26 ± 0.03
Sturgeon River	Male	69	104–145	0.46 ± 0.03
	Female	16	122–163	0.52 ± 0.08
Menominee River	Male	37	94–129	0.84 ± 0.07
	Female	26	121–146	0.93 ± 0.08
St. Clair River	Male	11	113–147	1.03 ± 0.24
	Female	29	126–165	1.15 ± 0.13
Lake Winnebago	Male	4063	96–178	0.79 ± 0.01
	Female	640	123–201	0.86 ± 0.02

**FIGURE 2:**
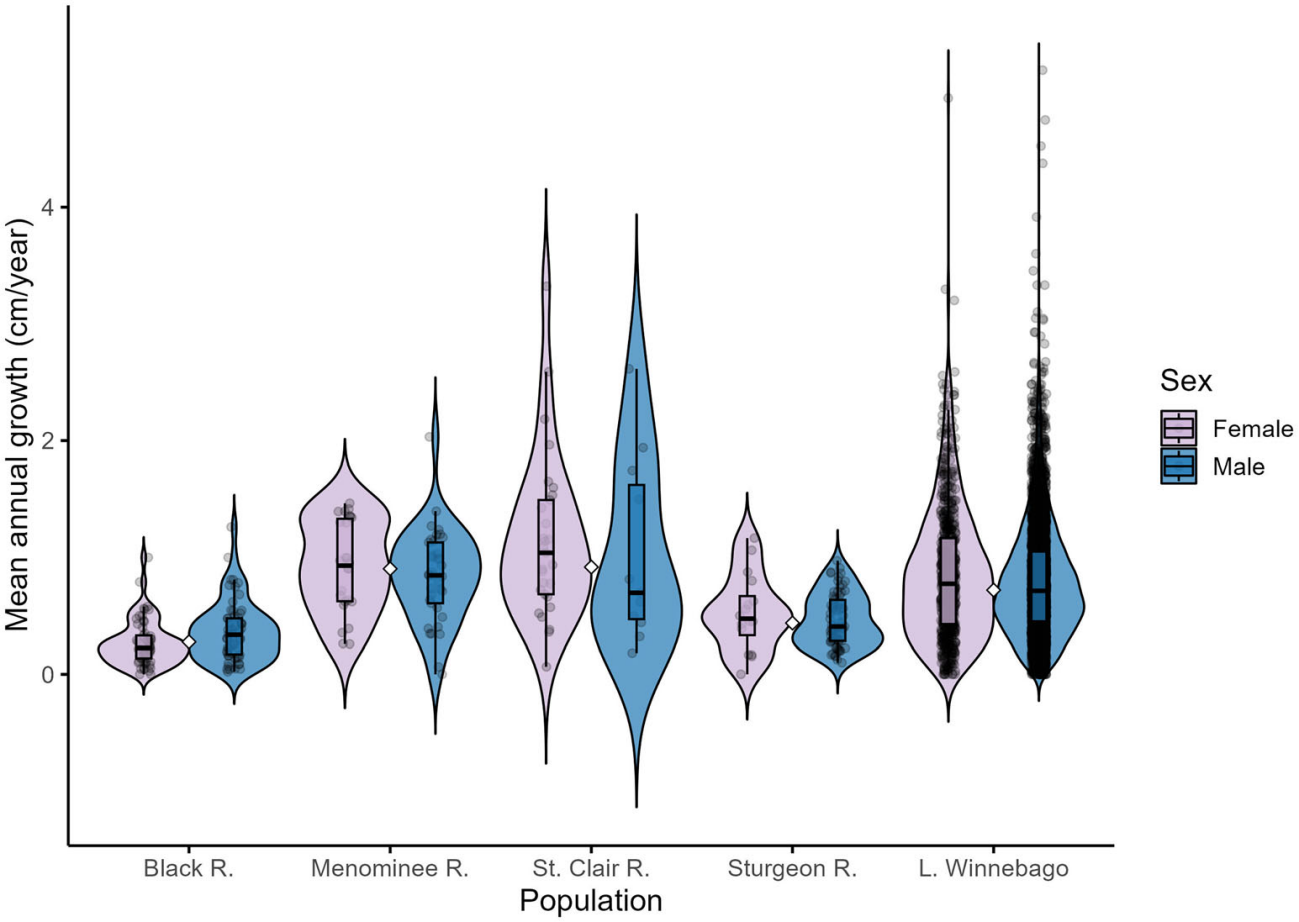
Distribution of annual growth rates by population and sex based on capture–mark–recapture data for individual lake sturgeon with at least 3 years between captures.

Mean annual growth varied among fish within a population, among populations, and between males and females (Table 1, Figure 2). The ANCOVA model showed significant effects of initial length (*F*_1,4996_ = 189.38, *p* < 0.001), sex (*F*_1,4996_ = 94.98, *p* < 0.001) and population (*F*_4,4996_ = 38.32, *p* < 0.001) on mean annual growth but no indication of an interaction effect between sex and population (*F*_4,4996_ = 0.85, *p* = 0.49).

Males had an estimated mean growth of 0.65 ± 0.04 cm year^−1^ compared with 0.85 ± 0.04 cm year^−1^ for females based on estimated marginal means adjusting for initial length and across all populations. Post‐hoc comparisons among the five populations indicated significant differences in annual growth rate after adjusting for initial length and considering both sexes. Black River (0.42 ± 0.05 cm year^−1^) and Sturgeon River (0.51 ± 0.07 cm year^−1^) had the lowest growth rates of the five populations. Menominee River sturgeon (0.80 ± 0.06 cm year^−1^) were significantly higher in growth, but the St. Clair River population (1.11 ± 0.09 cm year^−1^) had the highest growth rates reported. Fish from the Lake Winnebago system population (0.89 ± 0.01 cm year^−1^) were statistically similar to both the Menominee and St. Clair River groups based on post‐hoc comparisons. Average annual female growth was faster than male growth for all populations except the Black River one (Table 1).

**FIGURE 3:**
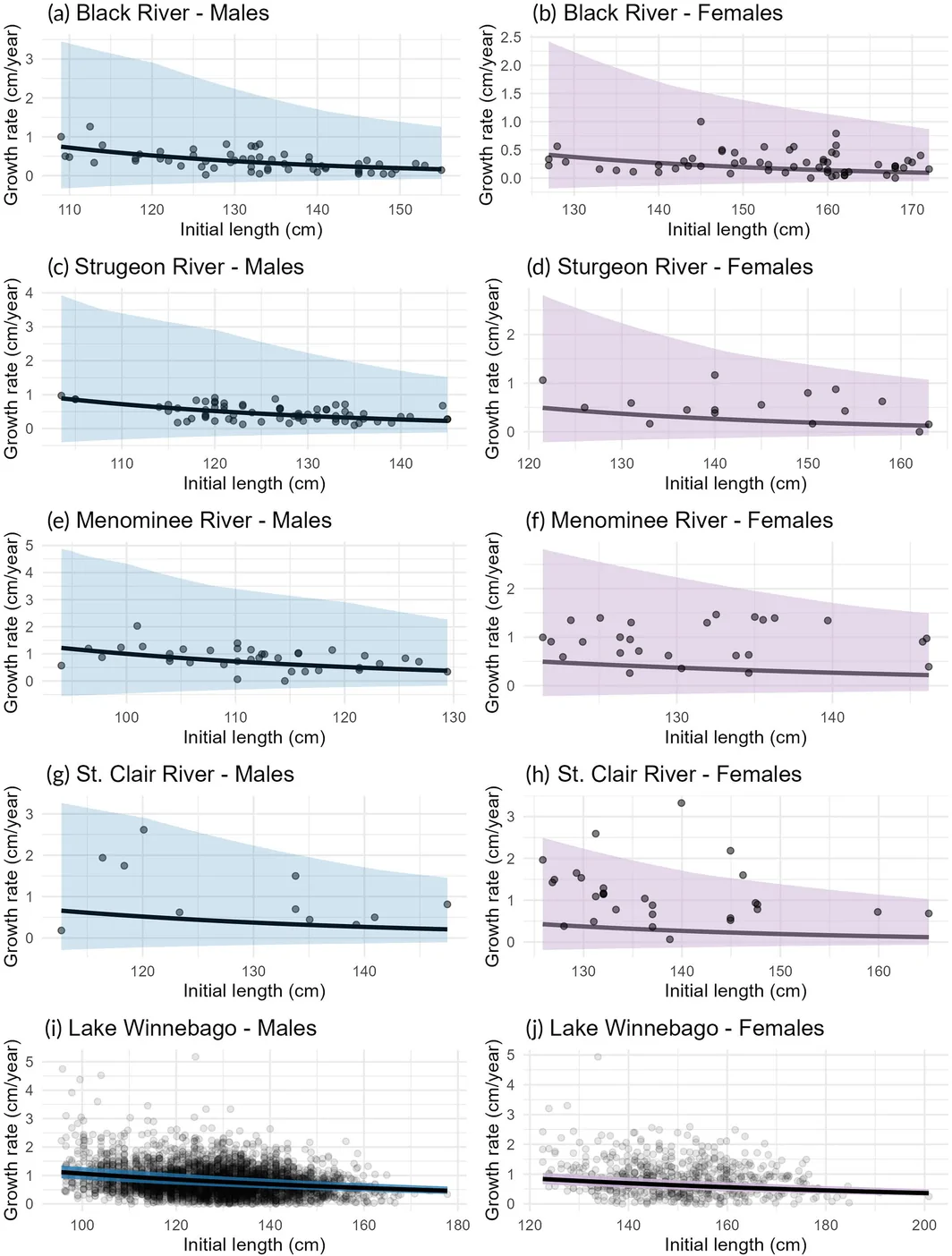
Sex‐specific mean annual growth rates (cm year^−1^) of adult lake sturgeon in relation to initial observed FL (cm) for fish from five populations. Points represent individual observations of mean annual growth between observation periods and solid black lines represent the predicted growth curve fit from a nonlinear least squares (NLS) regression with an exponential decay function. Shaded ribbons indicate 95% confidence intervals generated through 1000 simulations incorporating uncertainty in model parameter estimates (see Section 2).

Exponential curves and equations describing the relationship between size at initial capture and annual growth are shown in Figure 3 and Table 2 and indicated slower growth for the largest fish in each population regardless of sex. Slow growth was also estimated for some relatively small fish. Exponential curves describing growth rate as a function of FL at initial capture (see Equation 1) had similar shapes across populations, indicating that for both sexes growth rate slowed with increasing FL at initial capture (i.e., age). The *X* and *Z* parameters (Table 2) estimated from the exponential fit were then used as starting parameter values in the NLS estimation of growth rates for each population and sex (see Section 2).

**TABLE 2:** Summary of sample sizes, parameter estimates, and *R* ^2^ values for initial exponential regressions describing the mean annual growth rate (cm year^−1^) as a function of initial fork length (cm) for each of five lake sturgeon populations and both sexes.

Population	Males				Females			
	*N*	*X*	*Z*	*R* ^2^	*N*	*X*	*Z*	*R* ^2^
Black River	58	46.62	−0.04	0.34	58	5.24	−0.02	0.02
Sturgeon River	69	8.24	−0.02	0.24	16	1678.00	−0.06	0.13
Menominee River	37	18.66	−0.03	0.16	26	1.26	−0.003	0.0002
St. Clair River	11	5.57	−0.02	0.16	29	7.09	−0.02	0.05
Lake Winnebago	4063	3.02	−0.01	0.05	640	5.07	−0.01	0.05

*Note*: These fits were used to characterize the overall size‐growth relationship (see Equation 1). The parameters listed here are *X*, a scaling parameter that estimates annual growth at smaller body sizes, and *Z*, which describes the rate at which growth declines with increasing body length. The reported *R* ^2^ values indicate the variation in mean annual growth explained by this exponential relationship for each population and sex.

The NLS exponential models describing the relationship between mean annual growth and initial FL generally provided a good fit for most lake sturgeon population–sex groups, with models achieving convergence for all groups (Figure 3; Table 3). Model fits were strongest in the Winnebago population. This was the only population showing statistically significant differences from the null hypothesis for both the *X* and *Z* parameters for males and females (see Table 3 for model results by population and sex). Results may be related to the larger sample sizes for this population relative to the four other populations (Table 2). Among the four other populations, patterns were more variable. Black River and Sturgeon River males showed clear and statistically supported declines in growth with increasing size. Growth patterns for females in these systems were weaker and statistically non‐significant. Menominee River males exhibited a marginally significant relationship, but the model fit for females was weak overall. In the St. Clair River, growth models for both sexes had wide confidence intervals and were statistically non‐significant, likely due to smaller sample sizes. Overall, males tended to exhibit stronger size‐dependent growth declines than females. The strength and precision of fitted models were positively associated with sample size, especially evident in the Winnebago system.

**TABLE 3:** Parameter estimates from nonlinear least squares (NLS) fits of the exponential growth model (see Equation 1) describing mean annual growth rate (cm year^−1^) as a function of initial fork length (cm) for five lake sturgeon populations and each sex.

Population	Sex	*X*	*Z*	Residual error
Black River	Male	27.19 ± 21.15, *p* = 0.20	**−0.033 ± 0.0063, *p* < 0.001**	0.21, df = 56
	Female	0.92 ± 1.06, *p* = 0.39	−0.0081 ± 0.0076, *p* = 0.29	0.19, df = 56
Sturgeon River	Male	12.99 ± 8.56, *p* = 0.13	**−0.027 ± 0.0054, *p* < 0.001**	0.20, df = 67
	Female	6.72 ± 11.58, *p* = 0.57	−0.018 ± 0.012, *p* = 0.17	0.31, df = 14
Menominee River	Male	8.72 ± 8.04, *p* = 0.29	**−0.021 ± 0.0085, *p* = 0.02**	0.37, df = 35
	Female	1.02 ± 1.58, *p* = 0.52	−0.00075 ± 0.012, *p* = 0.95	0.40, df = 24
St. Clair River	Male	22.95 ± 61.57, *p* = 0.72	−0.024 ± 0.02, *p* = 0.29	0.77, df = 9
	Female	9.39 ± 17.63, *p* = 0.60	−0.015 ± 0.014, *p* = 0.28	0.70, df = 27
Lake Winnebago	Male	**3.10 ± 0.27, *p* < 0.001**	**−0.01 ± 0.00070, *p* < 0.001**	0.49, df = 4061
	Female	**5.02 ± 1.58, *p* = 0.002**	**−0.012 ± 0.0021, *p* < 0.001**	0.57, df = 638

*Note*: Values in bold were statistically significantly different from the null hypothesis. In this model, *X* is a scaling parameter estimating the annual growth increments at smaller body sizes and *Z* estimates the rate at which growth declines with length. Estimates, standard errors, *t*‐statistics, and associated *p*‐values are shown for each parameter, along with residual standard error of the model.

Despite variability in model fit and statistical significance among the population and sex groups, particularly females, the decline in growth rate with increasing size was consistently observed across all groups. Even though fitted parameters for some groups (e.g., females in Black River and St. Clair River) were statistically non‐significant, the direction of the relationship was uniformly negative and aligned with predicted patterns of growth for sturgeon. Furthermore, model predictions remained stable across the observed size ranges, and bootstrapped confidence intervals provided a robust means of incorporating parameter uncertainty into age estimates. Therefore, these growth models were deemed appropriate for use in back‐calculation estimates from observed lengths for sturgeon, particularly when paired with maturity‐based priors (length and age of maturity; see Section 2) that also informed the age estimations used here.

### 3.2 Estimated ages at size

**FIGURE 4:**
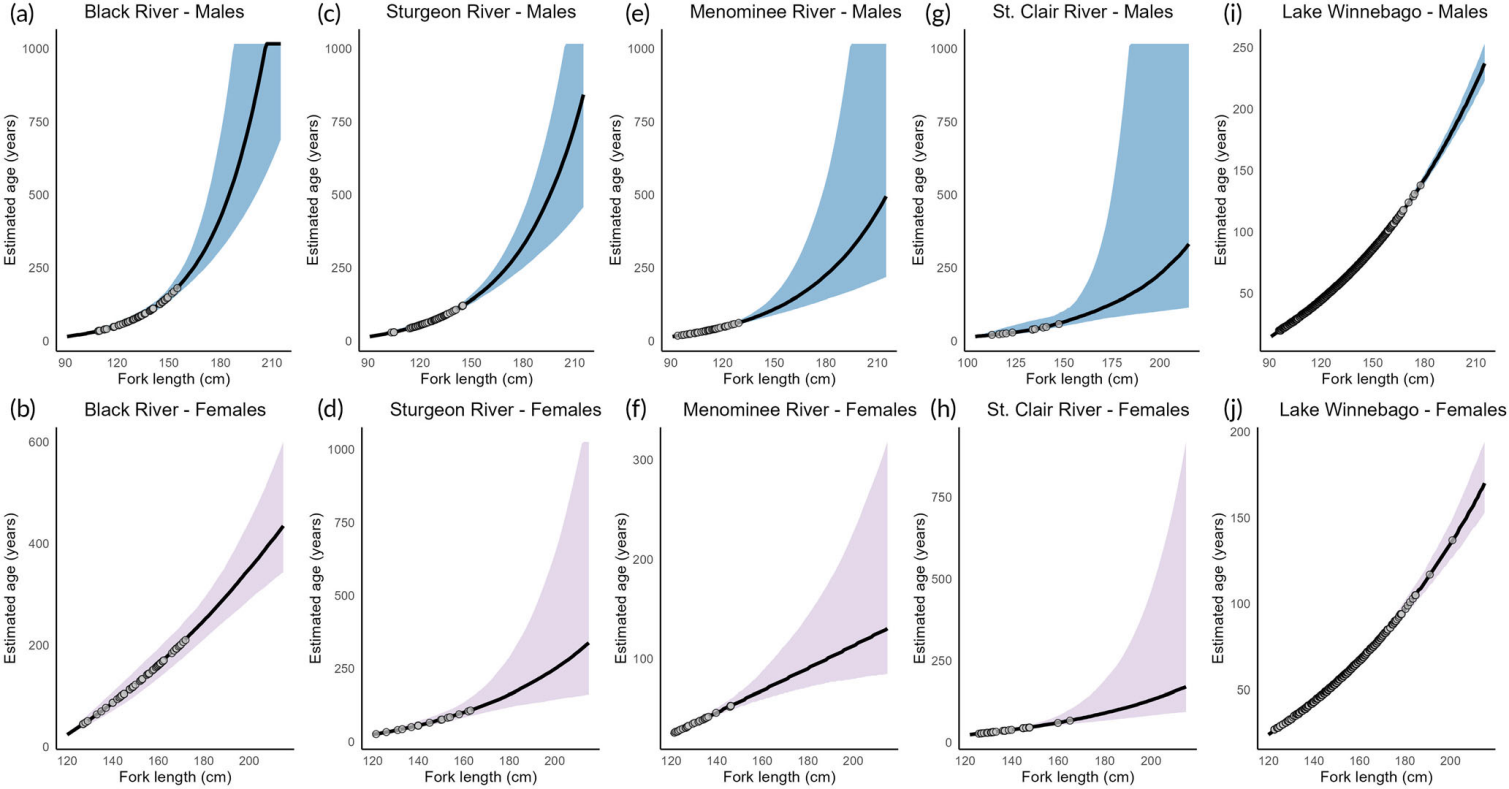
Estimated age (years) of adult lake sturgeon as a function of FL from five populations and for both sexes. Individual points represent fish for which mean annual growth rates were available and used to backcalculate age using an iterative subtraction of predicted growth increments until reaching the expected size and age at maturity (see Section 2). Solid black lines represent median predicted age‐at‐length relationships, and shaded ribbons indicate 95% confidence intervals generated from bootstrap resampling of the exponential growth relationship. Please note that due to the variability in age estimates (*y*‐axes), the panels do not represent standardized ranges in the data presented.

Using the population‐ and sex‐specific *X* and *Z* parameters estimated from Equation (1), age‐at‐length was calculated via the recursive procedure described in Equation (2), and uncertainty was incorporated using a bootstrapping procedure (see Section 2 for details). Because *X* determines the scaling of annual growth increments and *Z* controls the rate at which growth declines with size, these parameters directly governed the number of iterative steps required to reach the maturity threshold and, consequently, the total age predicted for a given fork length. In general, age estimates increased nonlinearly with length, with steeper age‐length curves typically observed in females (Figure 4). Across populations, projected ages at 160 cm for males ranged from 87 to 215 years and ranged from 90 to 248 years for 180 cm FL females (Table 4). Narrower confidence intervals were observed in populations with larger sample sizes and broader size distributions. Predicted median ages for the largest observed males ranged from 94 to 435 years among populations. Female predicted median ages for the largest observed fish ranged from 73 to 292 years (Table 5), reflecting differences in modelled size‐dependent growth trajectories. Across both sexes, individuals from the St. Clair River were consistently predicted to reach large sizes at younger ages relative to fish from the other populations.

**TABLE 4:** Estimated median age and 95% confidence intervals for lake sturgeon from five populations.

Population	Sex	Median age (years)	95% CI age (years)
Black River	Male (160 cm FL)	215	184–272
	Female (180 cm FL)	248	210–295
Sturgeon River	Male (160 cm FL)	189	165–220
	Female (180 cm FL)	161	120–292
Menominee River	Male (160 cm FL)	140	107–231
	Female (180 cm FL)	90	70–143
St. Clair River	Male (160 cm FL)	87	63–192
	Female (180 cm FL)	90	67–190
Lake Winnebago	Male (160 cm FL)	103	102–105
	Female (180 cm FL)	97	93–102

*Note*: Age estimates were made for male lake sturgeon at 160 cm fork length (FL) and female lake sturgeon at 180 cm FL. Ages were derived using a recursive backcalculation procedure (see Equation 2), in which population‐ and sex‐specific growth parameters were used to iteratively subtract predicted annual growth increments from a given fork length until the sexual maturity age threshold was reached. Confidence intervals were generated from 1000 bootstrap resamples of the growth model, refitting *X* and *Z* for each resample and recalculating age‐at‐length to propagate parameter uncertainty.

**TABLE 5:** Sex‐specific age‐at‐length estimates (median and 95% confidence interval) for lake sturgeon at (i) the minimum and maximum fork lengths (FL, cm) included in the capture–mark–recapture (CMR) growth models and (ii) the maximum observed fork length for each of five study populations.

Population	Sex	Minimum CMR			Maximum CMR			Maximum observed		
		FL (cm)	Median age (years)	95% CI	FL (cm)	Median age (years)	95% CI	FL (cm)	Median age (years)	95% CI
Black River	Male	109	34	28–42	155	181	158–222	181	435	318–737
	Female	127	45	39–54	172	212	178–253	189	292	246–353
Sturgeon River	Male	104	29	27–35	145	121	112–134	158	178	157–206
	Female	122	27	26–29	163	108	89–151	175	144	112–243
Menominee River	Male	94	18	17–19	129	62	57–70	163	151	112–261
	Female	121	26	25–26	146	53	49–60	165	74	63–100
St. Clair River	Male	113	21	18–33	147	60	47–93	163	94	67–210
	Female	126	27	27–28	165	68	56–101	169	73	59–119
Lake Winnebago	Male	95	19	19–19	178	139	135–142	181	145	141–150
	Female	122	26	26–26	201	137	127–151	210	158	143–178

*Note*: Ages were calculated using a recursive backcalculation procedure (see Equation 2) to iteratively subtract predicted annual growth increments from a given fork length until the sexual maturity threshold was reached. Confidence intervals were derived from 1000 bootstrap resamples of the growth model, refitting *X* and *Z* for each resample to propagate parameter uncertainty into age estimates.

## 4 DISCUSSION

### 4.1 Growth

Our results demonstrated that sexually mature male and female adult lake sturgeon mean annual growth is slow and consequently, longevity was greater than current estimates based on fin‐spine estimated ages. Exponential curves fit to empirical data indicated growth slowed as fish grew larger (and older) and growth was slowest for the largest fish in each population. The observed growth patterns were broadly consistent with expectations based on the von Bertalanffy growth model (Fabens, 1965), with declining growth rates as size increased. However, rather than estimating an asymptotic length parameter (*L*_∞_), the exponential model used here estimated growth increments and the decline in growth as a function of length. Under this formulation, growth progressively declines and, as a result, approaches zero at large body sizes, effectively providing an asymptote. The relatively slow growth rates at larger sizes that we observed are consistent with a shift in energy allocation from somatic growth toward reproduction (Heikkonen, 2012). As an illustration of the slow post‐maturity growth described above, a male lake sturgeon initially captured in the Sturgeon River in 1990 was 117 cm FL at initial capture and when most recently recaptured in 2024 (34 years between capture events) had only grown 7 cm and was 124 cm FL, an average annual increase in length of 0.21 cm year^−1^. In Black River, a female lake sturgeon captured in 2012 was 127 cm FL and when recaptured in 2021 was 129 cm FL, a mean annual growth rate of 0.22 cm year^−1^. Given these slow growth rates even for relatively small fish, extreme longevity for a species that routinely reaches lengths of 180 cm FL should be expected.

Mean annual growth rates varied among populations and may be related to latitude as previous authors have suggested. Power and McKinley (1997) examined data for 16 lake sturgeon populations across the species range and noted that, for a given age, fish from northern populations were smaller than southern populations. Growth differences were attributed to differences in the length of the growing season north to south. Habitat characteristics such as overall productivity and energy availability may also explain some of the variation. Fortin et al. (1996) also documented differences in growth and condition among 32 lake sturgeon populations and attributed differences to latitude. However, differences were also evident from east to west and were related to pH and conductivity. Our data are consistent with reports by Power and McKinley (1997) in that the St. Clair River fish, the southern‐most population examined here, had the fastest growth compared with the northern populations. Bioenergetics studies on lake sturgeon populations across the species range may provide valuable insights into mechanisms driving the observed growth differences.

Mean annual growth was slowest for the Black River and Sturgeon River populations. Outside of the spawning season, lake sturgeon in the Sturgeon River population occupy near‐shore waters of Lake Superior, a large oligotrophic lake, which may explain the slow growth exhibited by Sturgeon River fish. The Black River population had the slowest mean annual growth among the populations we examined despite Black Lake being more productive than Lake Superior. Black Lake is relatively small (~2500 ha), and fish are confined to the lake and may be resource limited. The density of lake sturgeon may limit food availability and result in slow growth relative to the other populations examined. The Lake Winnebago, Menominee and St. Clair River populations occupy relatively productive habitats and had the highest growth rates of the populations we studied. It is worth noting that individual fish growth rates likely vary through time as a function of resource availability. However, whether individual fish growth variation through time affects longevity is impossible to determine given our data. Interannual variation in growth rate resulting from varying resource availability (i.e., high growth in resource‐abundant years) would result in greater average annual growth rate estimates and consequently reduced age (longevity) estimates for a given fish length as compared with steady and slow growth through time.

### 4.2 Longevity

Our results show lake sturgeon longevity may be similar to other long‐lived fish taxa. Extreme fish longevity (400+ years) has been documented in Greenland Shark (Nielsen et al., 2016) and for several species of rockfish, including Rougheye Rockfish (*Sebastes aleutianus*) with an estimated age of 205 for a fish from the north Pacific (Kolora et al., 2021; Munk, 2001). Extreme longevity in fishes has been related to cold environments (Mangel & Abrahams, 2001). Long‐lived Greenland Shark and rockfish live in the northern oceans and at depths where water temperatures are consistently cold. However, lake sturgeons live in mid‐latitude freshwater habitats and are subject to annual seasonal changes in water temperature from near freezing to temperatures >20°C. Extreme longevity in fishes may therefore not be strictly tied to the temperature of the habitat or to marine habitats but may depend on other factors like evolutionary history (Beverton, 1987). As noted earlier, sturgeons are an ancient group of fish (Bemis et al., 1997). The coelacanth, another phylogenetically basal species, is also known to live to great age (Fricke et al., 2011). Sturgeons are in one (Chondrostei) of only two subclasses of fishes (Cladistia is second), which fossil evidence reveals evolved much earlier than, and separate from, the subclass Neopterygii or Modern Bony Fishes (Teleostei) (Helfman et al., 2009).

One physiological mechanism lake sturgeon may employ to increase longevity is torpor. Torpor is known to increase longevity in multiple mammal and bird species (Nowack et al., 2017) and has been recently documented in shortnose sturgeon (*Acipenser brevirostrum*). During winter, Andrews et al. (2020) used side‐scan sonar and observed groups of shortnose sturgeon over 4 years of winter surveys in the St. John River, New Brunswick, Canada. The groups were closely aggregated on the river bottom and located in the same areas each winter (January–March). Subsequent work used an underwater camera to observe ‘torpor’ in the aggregated shortnose sturgeon (Andrews et al., 2025). Aggregations of shortnose sturgeon were observed resting motionless on the river bottom and remained motionless even when touched by the camera and LED light. Sturgeons at northerly latitudes may use winter torpor to conserve energy, which could also extend longevity (Nowack et al., 2017). Of the populations we studied, the Sturgeon River is at a similar latitude to the St. John River and post‐spawning lake sturgeon are known to move out of the river and disperse widely in near‐shore waters of Lake Superior (Auer, 1999). If Sturgeon River lake sturgeon exhibit torpor, they may seek sheltered bays during winter. Further research into the winter ecology of lake sturgeon is warranted to determine if fish enter a state of torpor.

The observed differences in growth among populations resulted in differences in estimated longevity as well. What is not clear from our data is whether differences in longevity across populations represent life‐history trait variation among populations or are an artefact of sample size and historical exploitation levels. Blanck and Lamouroux (2007) examined life‐history traits for 25 European fish species and found that intraspecific variation in six life‐history traits, including growth and longevity, was related to latitude; longevity was greater for populations at higher latitudes. This is consistent with our results and suggests that life‐history traits may vary with latitude in lake sturgeon.

### 4.3 Survival

Underestimation of true ages can lead to underestimation of annual survival and consequent mismanagement of populations (e.g., setting allowable harvest rates too high). Lake sturgeon harvest fisheries typically target larger, older fish, although regulations vary. For example, the Lake Winnebago, WI, spear fishery has a minimum length limit of 91 cm and the hook and line harvest fishery has a minimum length limit of 152 cm. In contrast, the Black Lake, MI, fishery lacks a minimum length limit allowing harvest of small fish. Accurately assigning age to sexually mature lake sturgeon from pectoral fin‐spine annuli counts, the most used method for sturgeon species (e.g., Rien & Beamesderfer, 1994; Whiteman et al., 2004), is widely accepted to be challenging (Hessenauer et al., 2018; Rossiter et al., 1995), particularly as fish age and body size increase. Accordingly, underestimation of true age (and survival) can be substantial (Bruch et al., 2009). For this reason, Bruch et al. (2009) advised caution when using pectoral fin‐spine derived ages for setting management policy for lake sturgeon. Our results suggest lake sturgeon routinely live much longer than currently accepted longevity and consequently must have high annual survival as well (Hewitt and Hoenig, 2005).

High annual adult survival independent of fin‐spine determined ages has been estimated for three lake sturgeon populations in the Great Lakes. Annual survival for the St. Clair River adult lake sturgeon population was estimated from acoustic tagging and telemetry data from 2012 to 2019 using a Cormack‐Jolly‐Seber (CJS) model where fish were marked with acoustic tags and ‘recaptured’ over the course of the 7‐year study via detection by stationary acoustic receivers throughout the system. Annual survival was determined to be 0.97 across all years of study (Colborne et al., 2021). Sex‐specific annual adult survival for the Black Lake population has been estimated based on spawning run mark–recapture data collected from 2001 to 2024 and a modified CJS model that accounts for spawning periodicity (Pledger et al., 2013). Annual survival rates estimated in 2024 were 0.97 and 0.96 for females and males respectively. Annual survival rates estimated for lake sturgeon in Black Lake would likely be slightly higher but there is a recreational fishery with an annual allowable exploitation rate of up to 0.012. In addition, although rare, emigration from Black Lake over Alverno Dam on the lake outlet is known to occur and fish that emigrate are unable to return to Black Lake. Shrovnal et al. (2025) compared annual survival for the Lake Winnebago lake sturgeon population using age‐based catch‐curve analysis and capture–recapture methods and concluded capture–recapture methods resulted in higher estimates of annual survival than catch‐curve analysis; annual survival estimates were 0.976 and 0.960 for females and males, respectively. Like Black Lake, estimated annual survival would be higher but there is a recreational fishery on Lake Winnebago with an annual exploitation rate of up to 0.05 of the adult population (Shrovnal et al., 2025). Finally, catch‐curve analysis was used to estimate annual survival of 0.95 in Namakan Reservoir (Shaw et al., 2012) and Rainy River (Adams Jr et al., 2006), both in Minnesota. High annual adult survival thus appears to be consistent among populations across the species range.

### 4.4 Study limitations

Differences in sampling effort and methods among the five study populations may explain some of the differences in estimated growth rates and longevity estimates. Sample size was much lower for the St. Clair River population. In addition, the distribution of fish lengths used in estimation of growth rates was not equal among the five populations. Differences in the distribution of fish lengths were most obvious for the Menominee River population. The maximum size of male and female lake sturgeon that met the criteria of at least 3 years between capture events was 129 and 146 cm FL, respectively. These values are much smaller than for the other four populations and may explain in part why mean annual growth of Menominee River lake sturgeon was intermediate and faster compared with Black and Sturgeon River fish. Additional data from larger and likely slower growing fish from the Menominee River population may lead to a slower mean annual growth rate for the population. The observed differences among populations are likely due to different sampling efforts and methods used among populations as well as differences in demographic characteristics among populations. Although sampling methods differed, it is unlikely that different sampling methods selected for fish growing at faster versus slower rates within or among populations.

A notable limitation of our approach is that we were unable to precisely estimate age for individual fish. Rather, our approach predicts a range of ages for fish of particular length and sex. Nevertheless, we believe our statistically based approach provides more accurate estimates of sex‐specific lake sturgeon growth rates and expected longevity across the five populations studied here than could be achieved by counting annuli in fin‐ray cross sections because annuli counts from fin‐ray cross sections are known to underestimate the true age of lake sturgeon after fish reach sexual maturity and growth slows (Bruch et al., 2009).

Age determination in fishes should be validated using fish of known age or other methods, including bomb radiocarbon dating (Campana, 2001; Devries & Frie, 1996). The longevity of lake sturgeon makes it difficult to use known age fish for validation. A fish that could be confidently aged as a juvenile of known age or a stocked fish would need to be marked and subsequently recaptured multiple decades to hundreds of years later to confirm longevity in the range we estimated here. Perhaps radiocarbon dating could be used to at least estimate a range for lake sturgeon longevity as was done for the Greenland Shark (Nielsen et al., 2016).

### 4.5 Management implications

Regardless of population and growth rate, ages estimated for 160 and 180 cm FL male and female lake sturgeon were considerably greater than previous fin‐spine derived age estimates for lake sturgeon, suggesting lake sturgeon may routinely attain greater ages than previously estimated. This result has important implications for future lake sturgeon management because the annual survival rates associated with great age must be high and consequently harvest limits for lake sturgeon should be conservative to ensure population sustainability (Boreman, 1997; Congdon et al., 1993). It is also apparent from our results that there is considerable among‐population variability in growth rates and projected longevity estimates that should also be accounted for when formulating lake sturgeon management policy. Finally, our results suggest that longevity for other sturgeon species should be critically examined relative to current management policies, particularly for species and populations that are subject to harvest fisheries.

## AUTHOR CONTRIBUTIONS

E.A.B. contributed to conceptualization of manuscript idea, conceptualization of research topic, funding acquisition, investigation, methodology, data curation, data analysis, data visualization, and writing‐original draft, review, and editing. S.F.C. contributed to funding acquisition, investigation, data curation, data analysis, data visualization and writing‐review, and editing. N.A.A. contributed to funding acquisition, investigation, data curation, and writing‐review and editing. K.T.S. contributed to funding acquisition, investigation, data curation, data analysis, and writing‐review and editing. D.L.L. contributed to investigation, data curation, and writing‐review and editing. A.S.B. contributed to funding acquisition, investigation, data curation, and writing‐review and editing. R.M.B. contributed to funding acquisition, investigation, data curation, and writing‐review and editing. M.H.S. contributed to funding acquisition, investigation, data curation, and writing review, and editing. M.C.D. contributed to funding acquisition, investigation, data curation, and writing‐review and editing.

## FUNDING INFORMATION

We received grants from the Great Lakes Fishery Trust, U.S. Fish and Wildlife Service, AgBioResearch, Michigan State University, National Marine Fisheries Service, Wisconsin Department of Natural Resources Sturgeon Fund, WE Energies Mitigation Enhancement Fund, Upper Peninsula Power Company, and the Michigan Department of Natural Resources to support the collection of data presented here.

## CONFLICT OF INTEREST STATEMENT

The authors declare no conflicts of interest.

## Data Availability

The data that support the findings of this study are openly available in dryad at https://datadryad.org/, reference number DOI https://doi.org/10.5061/dryad.ns1rn8q5r.

## References

[jfb70478-bib-0001] Adams, W. E., Jr. , Kallemeyn, L. W. , & Willis, D. W. (2006). Lake sturgeon population characteristics in rainy Lake, Minnesota and Ontario. Journal of Applied Ichthyology, 22(2), 97–102. 10.1111/j.1439-0426.2006.00725.x

[jfb70478-bib-0002] Andrews, S. N. , O' Sullivan, A. M. , Linannsaari, T. , & Curry, A. (2025). Using rapid and repeatable side scan sonar methods for a second assessment of the shortnose sturgeon (*Acipenser brevirostrum*) population in the Saint John River, New Brunswick, Canada. Proceedings of the Nova Scotian Institute of Science, 54(1), 135–166. 10.15273/pnsis.v54i1.12646

[jfb70478-bib-0003] Andrews, S. N. , O'Sullivan, A. M. , Helminen, J. , Arluison, D. F. , Samways, K. M. , Linnansaari, T. , & Curry, R. A. (2020). Development of active numerating side‐scan for a high‐density overwintering location for endemic shortnose sturgeon (*Acipenser brevirostrum*) in the Saint John River, New Brunswick. Diversity, 12(1), 23. 10.3390/d12010023

[jfb70478-bib-0004] Auer, N. A. (1996). Importance of habitat and migration to sturgeons with emphasis on lake sturgeon. Canadian Journal of Fisheries and Aquatic Sciences, 53(S1), 152–160. 10.1139/f95-276

[jfb70478-bib-0005] Auer, N. A. (1999). Population characteristics and movements of lake sturgeon in the Sturgeon River and Lake Superior. Journal of Great Lakes Research, 25(2), 282–293. 10.1016/S0380-1330(99)70737-9

[jfb70478-bib-0006] Baker, E. A. , & Borgeson, D. J. (1999). Lake sturgeon abundance and harvest in Black Lake, Michigan, 1975–1999. North American Journal of Fisheries Management, 19(4), 1080–1088. 10.1577/1548-8675(1999)019<1080:LSAAHI>2.0.CO;2

[jfb70478-bib-0007] Bemis, W. E. , Findeis, E. K. , & Grande, L. (1997). An overview of Acipenseriformes. Environmental Biology of Fishes, 48, 25–71. 10.1023/A:1007370213924

[jfb70478-bib-0008] Beverton, R. J. H. (1987). Longevity in fish: Some ecological and evolutionary considerations. In A. D. Woodhead & K. H. Thompson (Eds.), Evolution of longevity in animals. Springer. 10.1007/978-1-4613-1939-9_12 3435385

[jfb70478-bib-0009] Blanck, A. , & Lamouroux, N. (2007). Large‐scale intraspecific variation in life‐history traits of European freshwater fish. Journal of Biogeography, 34(5), 862–875. 10.1111/j.1365-2699.2006.01654.x

[jfb70478-bib-0010] Boreman, J. (1997). Sensitivity of north American sturgeons and paddlefish to fishing mortality. Sturgeon Biodiversity and Conservation, 48, 399–405. 10.1023/A:1007345806559

[jfb70478-bib-0011] Briggs, A. S. , Boase, J. C. , Chiotti, J. A. , Hessenauer, J. M. , & Wills, T. C. (2019). Retention of loop, monel, and passive integrated transponder tags by wild, free‐ranging Lake sturgeon (*Acipenser fulvescens* Rafinesque, 1817). Journal of Applied Ichthyology, 35(3), 629–635. 10.1111/jai.13905

[jfb70478-bib-0012] Bruch, R. M. (2008). Modeling the population dynamics and sustainability of Lake Sturgeon in the Winnebago system, Wisconsin. Dissertation, University of Wisconsin‐Milwaukee.

[jfb70478-bib-0013] Bruch, R. M. , Campana, S. E. , Davis‐Foust, S. L. , Hansen, M. J. , & Janssen, J. (2009). Lake sturgeon age validation using bomb radiocarbon and known‐age fish. Transactions of the American Fisheries Society, 138, 361–372. 10.1577/T08-098.1

[jfb70478-bib-0014] Campana, S. E. (2001). Accuracy, precision and quality control in age determination, including a review of the use and abuse of age validation methods. Journal of Fish Biology, 59(2), 197–242. 10.1111/j.1095-8649.2001.tb00127.x

[jfb70478-bib-0015] Chiotti, J. A. , Boase, J. C. , Briggs, A. S. , Davis, C. , Drouin, R. , Hondorp, D. W. , Mohr, L. , Roseman, E. F. , Thomas, M. V. , & Wills, T. C. (2023). Lake Sturgeon population trends in the St. Clair–Detroit River system, 2001–2019. North American Journal of Fisheries Management, 43(4), 1066–1080. 10.1002/nafm.10917

[jfb70478-bib-0016] Colborne, S. F. , Hayden, T. A. , Holbrook, C. M. , Hondorp, D. W. , & Krueger, C. C. (2021). Lake sturgeon (*Acipenser fulvescens*) annual adult survival estimated from acoustic telemetry. Journal of Great Lakes Research, 47(2021), 1814–1823. 10.1016/j.jglr.2021.08.007

[jfb70478-bib-0017] Congdon, J. D. , Dunham, A. E. , & van Loben Sels, R. C. (1993). Delayed sexual maturity and demographics of Blanding's turtles (*Emydoidea blandingii*): Implications for conservation and management of long‐lived organisms. Conservation Biology, 7(4), 826–833. 10.1046/j.1523-1739.1993.740826.x

[jfb70478-bib-0018] Das, M. (1994). Age determination and longevity in fishes. Gerontology, 40(2–4), 70–96. 10.1159/000213580 7926859

[jfb70478-bib-0019] DeHaan, P. W. , Libants, S. V. , Elliott, R. F. , & Scribner, K. T. (2006). Genetic population structure of remnant lake sturgeon populations in the upper Great Lakes basin. Transactions of the American Fisheries Society, 135(6), 1478–1492. 10.1577/T05-213.1

[jfb70478-bib-0062] Devries, D. R. , & Frie, R. V. (1996). Determination of age and growth. In B. R. Murphy & D. W. Wills , (Eds.), Fisheries techniques (2nd ed., pp. 483–512). American Fisheries Society.

[jfb70478-bib-0020] Fabens, A. J. (1965). Properties and fitting of the von Bertalanffy growth curve. Growth, 29, 265–289. 5865688

[jfb70478-bib-0021] Fortin, R. , Dumont, P. , & Guénette, S. (1996). Determinants of growth and body condition of lake sturgeon (*Acipenser fulvescens*). Canadian Journal of Fisheries and Aquatic Sciences, 53(5), 1150–1156. 10.1139/f96-043

[jfb70478-bib-0022] Fricke, H. , Hissmann, K. , Froese, R. , Schauer, J. , Plante, R. , & Fricke, S. (2011). The population biology of the living coelacanth studied over 21 years. Marine Biology, 158, 1511–1522. 10.1007/s00227-011-1667-x

[jfb70478-bib-0023] Healy, K. , Ezard, T. H. , Jones, O. R. , Salguero‐Gómez, R. , & Buckley, Y. M. (2019). Animal life history is shaped by the pace of life and the distribution of age‐specific mortality and reproduction. Nature Ecology & Evolution, 3(8), 1217–1224. 10.1038/s41559-019-0938-7 31285573

[jfb70478-bib-0024] Heikkonen, R. D. S. J. (2012). Estimating age at first maturity in fish from change‐points in growth rate. Marine Ecology Progress Series, 450, 147–157. 10.3354/meps09565

[jfb70478-bib-0025] Helfman, G. S. , Collette, B. B. , Facey, D. E. , & Bowen, B. W. (2009). The diversity of fishes: Biology, evolution, and ecology. John Wiley & Sons.

[jfb70478-bib-0026] Hessenauer, J.‐M. , Utrup, B. E. , Briggs, A. S. , Wills, T. C. , & Thomas, M. V. (2018). Mark‐recapture validation of pectoral fin ray age estimation for lake sturgeon. North American Journal of Fisheries Management, 38, 1251–1257. 10.1002/nafm.10228

[jfb70478-bib-0061] Hewitt, D. A. , & Hoenig, J. M. (2005). Comparison of two approaches for estimating natural mortality based on longevity. Fishery Bulletin, 103(2), 433–437.

[jfb70478-bib-0059] Holey, M. E. , Baker, E. A. , Thuemler, T. F. , & Elliott, R. F. (2000). Research and assessment needs to restore Lake Sturgeon in the Great Lakes: Results of a workshop sponsored by the Great Lakes Fishery Trust. Great Lakes Fishery Trust.

[jfb70478-bib-0060] Isermann, D. A. , Raabe, J. K. , Easterly, E. G. , Schulze, J. C. , Porter, N. J. , Dembkowski, D. J. , Donofrio, M. C. , Kramer, D. R. , & Elliott, R. F. (2022). Lake sturgeon movement after trap and transfer around two dams on the Menominee River, Wisconsin–Michigan. Transactions of the American Fisheries Society, 151(5), 611–629.

[jfb70478-bib-0027] Kanefsky, J. , Smith, S. , & Scribner, K. T. (2022). Real‐time PCR‐based method for sex determination in lake sturgeon (*Acipenser fulvescens*). Diversity, 14(10), 839. 10.3390/d14100839

[jfb70478-bib-0028] Kerns, J. A. , & Lombardi‐Carlson, L. A. (2017). History and importance of age and growth information. In Age and growth of fishes: Principles and techniques (pp. 1–8). American Fisheries Society.

[jfb70478-bib-0029] Kolora, S. R. R. , Owens, G. L. , Vazquez, J. M. , Stubbs, A. , Chatla, K. , Jainese, C. , Seeto, K. , McCrea, M. , Sandel, M. W. , Vianna, J. A. , & Maslenikov, K. (2021). Origins and evolution of extreme life span in Pacific Ocean rockfishes. Science, 374(6569), 842–847. 10.1126/science.abg5332 34762458 PMC8923369

[jfb70478-bib-0030] Larson, D. L. , Brenden, T. O. , Baker, E. A. , & Scribner, K. T. (2025). Changes in lake sturgeon spawning periodicity is associated with prior reproductive effort. Scientific Reports, 15(1), 3783. 39885297 10.1038/s41598-025-87717-x PMC11782595

[jfb70478-bib-0031] MacKay, H. H. (1963). Fishes of Ontario (p. 300). Ontario Department of Lands and Forests. Bryand Press Ltd.

[jfb70478-bib-0032] MacNeil, M. A. , McMeans, B. C. , Hussey, N. E. , Vecsei, P. , Svavarsson, J. , Kovacs, K. M. , Lydersen, C. , Treble, M. A. , Skomal, G. B. , Ramsey, M. , & Fisk, A. T. (2012). Biology of the Greenland shark *Somniosus microcephalus* . Journal of Fish Biology, 80, 991–1018. 10.1111/j.1095-8649.2012.03257.x 22497371

[jfb70478-bib-0033] Mangel, M. , & Abrahams, M. V. (2001). Age and longevity in fish, with consideration of the ferox trout. Experimental Gerontology, 36(4–6), 765–790. 10.1016/S0531-5565(00)00240-0 11295513

[jfb70478-bib-0034] Munk, K. M. (2001). Maximum ages of groundfishes in waters off Alaska and British Columbia and considerations of age determination. Alaska Fishery Research Bulletin, 8(1), 12–21. 10.1016/S0531-5565(00)00240-0

[jfb70478-bib-0035] Nielsen, J. , Hedeholm, R. B. , Heinemeier, J. , Bushnell, P. G. , Christiansen, J. S. , Olsen, J. , Ramsey, C. B. , Brill, R. W. , Simon, M. , Steffensen, K. F. , & Steffensen, J. F. (2016). Eye lens radiocarbon reveals centuries of longevity in the Greenland shark (*Somniosus microcephalus*). Science, 353(6300), 702–704. 10.1126/science.aaf1703 27516602

[jfb70478-bib-0036] Nowack, J. , Stawski, C. , & Geiser, F. (2017). More functions of torpor and their roles in a changing world. Journal of Comparative Physiology B, 187(5), 889–897. 10.1007/s00360-017-1100-y PMC5486538 28432393

[jfb70478-bib-0037] Peterson, D. L. , Vecsei, P. , & Jennings, C. A. (2007). Ecology and biology of the lake sturgeon: A synthesis of current knowledge of a threatened North American Acipenseridae. Reviews in Fish Biology and Fisheries, 17, 59–76. 10.1007/s11160-006-9018-6

[jfb70478-bib-0038] Pledger, S. , Baker, E. , & Scribner, K. (2013). Breeding return times and abundance in capture–recapture models. Biometrics, 69(4), 991–1001. 10.1111/biom.12094 24152120

[jfb70478-bib-0039] Pollock, M. S. , Carr, M. , Kreitals, N. M. , & Phillips, I. D. (2015). Review of a species in peril: What we do not know about lake sturgeon may kill them. Environmental Review, 23, 30–43. 10.1139/er-2014-0037

[jfb70478-bib-0040] Power, I. , & McKinley, R. S. (1997). Latitudinal variation in lake sturgeon size as related to the thermal opportunity for growth. Transactions of the American Fisheries Society, 126(4), 549–558. 10.1577/1548-8659(1997)126<0549:LVILSS>2.3.CO;2

[jfb70478-bib-0041] Randall, J. E. , & Delbeek, J. C. (2009). Comments on the extremes in longevity in fishes, with special reference to the Gobiidae. Proceedings of the California Academy of Sciences, 60(10), 447.

[jfb70478-bib-0042] Rien, T. A. , & Beamesderfer, R. C. (1994). Accuracy and precision of white sturgeon age estimates from pectoral fin rays. Transactions of the American Fisheries Society, 123(2), 255–265. 10.1577/1548-8659(1994)123<0255:AAPOWS>2.3.CO;2

[jfb70478-bib-0043] Rossiter, A. , Noakes, D. L. G. , & Beamish, E. W. H. (1995). Validation of age estimation for the lake sturgeon. Transactions of the American Fisheries Society, 124(5), 777–781. 10.1577/1548-8659(1995)124<0777:VOAEFT>2.3.CO;2

[jfb70478-bib-0044] Scribner, K. T. , & Kanefsky, J. (2021). Molecular sexing of lake sturgeon. Journal of Great Lakes Research, 47(3), 934–936. 10.1016/j.jglr.2021.03.015

[jfb70478-bib-0045] Shaw, S. L. , Chipps, S. R. , Windels, S. K. , Webb, M. A. H. , McLeod, D. T. , & Willis, D. W. (2012). Lake sturgeon population attributes and reproductive structure in the Namakan Reservoir, Minnesota and Ontario. Journal of Applied Ichthyology, 28(2), 168–175. 10.1111/j.1439-0426.2011.01927.x

[jfb70478-bib-0046] Shrovnal, J. S. , Stadig, M. H. , Raabe, J. K. , & Isermann, D. A. (2025). Estimating mortality of Lake Sturgeon in the Lake Winnebago system using traditional age‐based approaches and capture–recapture models. North American Journal of Fisheries Management, 45, 616–632. 10.1093/najfmt/vqaf044

[jfb70478-bib-0047] Sulak, K. J. , & Randall, M. (2002). Understanding sturgeon life history: Enigmas, myths, and insights from scientific studies. Journal of Applied Ichthyology, 18, 519–528. 10.1046/j.1439-0426.2002.00413.x

[jfb70478-bib-0048] Taylor, A. A. , Larson, D. L. , Scribner, K. T. , Baker, E. A. , Halden, N. M. , & Anderson, W. G. (2025). Using fin ray elemental signatures and growth zone width to estimate onset of sexual maturity in lake sturgeon (*Acipenser fulvescens*). Canadian Journal of Fisheries and Aquatic Sciences, 82, 1–14. 10.1139/cjfas-2023-0279

[jfb70478-bib-0049] Thomas, M. V. , & Haas, R. C. (1999). Capture of lake sturgeon with setlines in the St. Clair River, Michigan. North American Journal of Fisheries Management, 19(2), 610–612. 10.1577/1548-8675(1999)019<0610:COLSWS>2.0.CO;2

[jfb70478-bib-0050] Thorson, T. B. , & Lacy, E. J., Jr. (1982). Age, growth rate and longevity of *Carcharhinus leucas* estimated from tagging and vertebral rings. Copeia, 1982, 110–116. 10.2307/1444275

[jfb70478-bib-0051] Thuemler, T. F. (1985). The lake sturgeon, *Acipenser fulvescens*, in the Menominee River, Wisconsin‐Michigan. Environmental Biology of Fishes, 14, 73–78. 10.1007/BF00001578

[jfb70478-bib-0052] Thuemler, T. F. (1997). Lake sturgeon management in the Menominee River, a Wisconsin‐Michigan boundary water. Environmental Biology of Fishes, 48, 311–317. 10.1023/A:1007325300585

[jfb70478-bib-0053] Tsepkin, Y. A. , & Sokolov, L. I. (1971). The maximum size and age of some sturgeons. Journal of Ichthyology, 11(3), 444–446.

[jfb70478-bib-0054] Use of Fishes in Research Committee (joint committee of the American Fisheries Society, the American Institute of Fishery Research Biologists, and the American Society of Ichthyologists and Herpetologists) . (2014). Guidelines for the use of fishes in research. American Fisheries Society.

[jfb70478-bib-0055] Vecsei, P. , Litvak, M. K. , Noakes, D. L. , Rien, T. , & Hochleithner, M. (2003). A noninvasive technique for determining sex of live adult North American sturgeons. Environmental Biology of Fishes, 68(4), 333–338.

[jfb70478-bib-0056] Whiteman, K. W. , Travnichek, V. H. , Wildhaber, M. L. , DeLonay, A. , Papoulias, D. , & Tillett, D. (2004). Age estimation for shovelnose sturgeon: A cautionary note based on annulus formation in pectoral fin rays. North American Journal of Fisheries Management, 24(2), 731–734. 10.1577/M03-090.1

[jfb70478-bib-0057] Withers, J. L. , Gorsky, D. , Biesinger, Z. , Einhouse, D. , Clancy, M. , Davis, L. , Karboski, C. , Legard, C. , Bruestle, E. , Markley, N. , & Roth, R. (2020). Age and growth of Niagara River Lake Sturgeon. Journal of Fish and Wildlife Management, 11(2), 634–643. 10.3996/102019-JFWM-085

[jfb70478-bib-0058] Young, J. L. , Bornik, Z. B. , Marcotte, M. L. , Charlie, K. N. , Wagner, G. N. , Hinch, S. G. , & Cooke, S. J. (2006). Integrating physiology and life history to improve fisheries management and conservation. Fish and Fisheries, 7(4), 262–283. 10.1111/j.1467-2979.2006.00225.x

